# Influence of Socioeconomic Status on Survival of Hepatocellular Carcinoma in the Ontario Population; A Population-Based Study, 1990–2009

**DOI:** 10.1371/journal.pone.0040917

**Published:** 2012-07-13

**Authors:** Nathaniel Jembere, Michael A. Campitelli, Morris Sherman, Jordan J. Feld, Wendy Lou, Stuart Peacock, Eric Yoshida, Murray D. Krahn, Craig Earle, Hla-Hla Thein

**Affiliations:** 1 Dalla Lana School of Public Health, University of Toronto, Toronto, Ontario, Canada; 2 Institute for Clinical Evaluative Sciences, Toronto, Ontario, Canada; 3 Toronto General Hospital, University Health Network/University of Toronto, Toronto, Ontario, Canada; 4 Liver Centre, Toronto Western Hospital, University Health Network/University of Toronto, Toronto, Ontario, Canada; 5 Canadian Centre for Applied Research in Cancer Control (ARCC), University of British Columbia, Vancouver, British Columbia, Canada; 6 University of British Columbia, Vancouver, British Columbia, Canada; 7 Toronto Health Economics and Technology Assessment Collaborative (THETA), Toronto, Ontario, Canada; 8 Departments of Medicine and Health Policy, Management and Evaluation and Faculty of Pharmacy, University of Toronto, Toronto, Ontario, Canada; 9 Ontario Institute for Cancer Research/Cancer Care Ontario, Toronto, Ontario, Canada; Broad Institute of Massachusetts Institute of Technology and Harvard University, United States of America

## Abstract

**Background:**

Research has shown that people from higher socioeconomic status (SES) have better hepatocellular carcinoma (HCC) survival outcomes, although no such research has been carried out in Canada. We aimed to assess if an association between SES and HCC survival existed in the Canadian context.

**Methodology/Prinicpal Findings:**

We conducted a population-based cohort study linking HCC cases identified in the Ontario Cancer Registry between 1990 and 2009 to administrative and hospital data. Logistic regression and chi-squared tests were used to evaluate associations between SES (income quintile) and covariates. The Kaplan-Meier method was used to estimate survival. Sequential analysis of the proportional-hazards models were used to determine the association between SES and HCC survival controlling for potential prognostic covariates. During the period 1990–2009**,** 5,481 cases of HCC were identified. A significant association was found between SES and curative treatment (p = 0.0003), but no association was found between SES and non-curative treatment (p = 0.064), palliative treatment (p = 0.680), or ultrasound screening (p = 0.615). The median survival for the lowest SES was 8.5 months, compared to 8.8 months for the highest SES group. The age- and sex-adjusted proportional-hazards model showed statistically significant difference in HCC survival among the SES groups, with hazard ratio 0.905 (95% confidence intervals 0.821, 0.998) when comparing highest to lowest SES group. Further adjustments indicated that potentially curative treatment was the likely explanation for the association between SES and HCC survival.

**Conclusions/Significance:**

Our findings suggest that a 10% HCC survival advantage exists for the higher SES groups. This association between SES and HCC survival is most likely a reflection of lack of access to care for low SES groups, revealing inequities in the Canadian healthcare system.

## Introduction

The incidence of hepatocellular carcinoma (HCC) has been increasing in Canada over the past several decades. [1], [2] Age-adjusted incidence of HCC has been found to increase 3.4% per year in males and 2.2% per year in females over the past 30 years, attributed to an increase in the incidence of viral hepatitis, immigration of people from countries of high viral hepatitis endemicity, obesity, and diabetes. [1], [2] Juxtaposed with the increase in HCC incidence has been an improvement in the screening, diagnosis, and treatment of HCC to improve HCC survival. [3]

Although there have been advances in cancer treatment, improvements in survival outcomes have not been equally distributed among all social classes. Studies have shown that people from the highest socioeconomic status (SES) have better survival outcomes compared to those in the lowest SES. [4], [5] Multiple theories have been proposed for the observed survival advantage for people from higher SES. It has been proposed that people from higher SES seek treatment earlier in the disease progression, whereas people from lower SES withhold from seeking for treatment until the cancer has become symptomatic and incurable. [6], [7], [8], [9] Other theories are that people from higher SES have better access to treatment and care, [10], [11] and that people from higher SES have lower levels of comorbidity, leading to lower cause-specific cancer mortality as well as unrelated deaths. [12]

Healthcare within Ontario, framed by the Canadian Health Act of 1985, [13] is founded upon the principles of universality and accessibility. Healthcare is publically administered through the Medicare program, to ensure all citizens have universal access to health services on a prepaid basis and alleviate any financial burden of healthcare on its citizens. [14] Reported research in Canada has been contradictory, with some studies reporting no cancer survival association with SES, [15], [16], [17], [18] while other studies report survival advantages of certain non-HCC cancers (such as breast and colon cancers) being influenced by the effects of SES. [12], [19], [20]

Our main objective of the study was to determine whether there was an association between SES and HCC survival in Ontario. We also sought to determine if the relationship between SES and HCC survival was confounded by comorbidity, screening, and treatment by socioeconomic class. Differences in results from our study and studies from countries without universal healthcare access may provide insight into the relationship between the lack of healthcare access and its effects on certain socioeconomic groups.

## Methods

### Ethics Statement

Ethics approval for the study was granted by the University of Toronto Health Sciences Research Ethics Board.

### Study Design

A population-based retrospective cohort study was conducted on all diagnosed cases of HCC in Ontario, between January 1, 1990 and December 31, 2009. Cases were identified from the Ontario Cancer Registry (OCR), a population-based tumor registry for Canada’s largest province (population ∼12 million) operated by Cancer Care Ontario. [21] Persons were followed from the day of diagnosis up until death or until to the end of the study period.

We used the International Statistical Classification of Disease and Related Health Problems (ICD-9) site code 155.0 (primary malignant neoplasm of the liver) and the International Classification of Diseases for Oncology, Third Edition (ICD-O-3) histology codes 8170–8175 to identify cases of primary liver cancer. Excluded individuals from the study were those who had death dates before or on diagnosis date, and people without a valid Ontario healthcare number.

### Data Sources

The OCR is a population-based cancer registry that collects data on incident cases of tumors in Ontario since 1964. [22] Data from multiple sources, including electronic reports from the Canadian Institute for Health Information (CIHI), paper reports from pathology departments, electronic reports from the nine Ontario Regional Cancer Centers and from the Princess Margaret Hospital, and electronic reports of all deaths of Ontario residents from the office of the Registrar General of Ontario based on Ontario Provincial death certificates with cancer as the underlying cause of death are probabilistically linked to compile incident cases of cancer in Ontario. [23] About 95% of all diagnosed cases of cancer are estimated to be captured by the OCR. [23] The overall quality of the OCR data has been found to be high; using chart abstraction as the gold standard, the OCR has been shown to be 97–100% complete in tumor morphology and year of diagnosis. [24]

The OCR was linked to the Ontario Health Insurance Plan (OHIP) database, CIHI discharge abstract database, the Ontario Drug Benefit (ODB) program database, and Canadian census data, to provide individual-level information on demographic, screening and treatment factors. The OHIP physician billing claims dataset contains service and diagnosis information for outpatient visits in Ontario. The CIHI discharge abstract database contains information pertaining to diagnosis and procedures for all acute and chronic care hospitalizations in Ontario. The ODB dataset contains information regarding prescription medications dispensed to all adults aged 65 and above and those receiving social assistance. The 1991, 1996, 2001, and 2006 Canadian census data were used to gather information on the socioeconomic variable of neighborhood income quintile. The 1991 Canadian census was used to link postal code to the neighborhood income quintile for people diagnosed from 1991–1993. The 1996, 2001, and 2006 Canadian census data were used to assign postal codes to neighborhood income quintile for people diagnosed during 1994–1998, 1999–2003, and 2004–2009, respectively.

### Data Linkage

Using encrypted healthcare numbers, surname, given name, date of birth, and sex, the cancer registry data was probabilistically linked to health administrative datasets. Record linkage was performed by the Institute for Clinical Evaluative Sciences using AUTOMATCH Generalized Record Linkage System software, [25] and then anonymized.

### Outcome Measure

The main outcome for our study was survival time after diagnosis. Date of HCC diagnosis and date of death were ascertained from the OCR to calculate the length of survival after diagnosis.

### Neighborhood Income Quintile

The OCR does not contain socioeconomic data, and therefore individual-level income could not be ascertained. Median household neighborhood income was instead used as a proxy variable for SES. Neighborhood quintiles were created by dividing the entire distribution of Ontariòs median neighborhood income into five quintiles. Individual median neighborhood household income was ascertained by linking OCR postal codes to the Canadian census enumeration area (maximum of 650 residences). The first quintile represents the lower 20% of neighborhoods with the poorest median neighborhood income. The fifth quintile represents the upper 20% of neighborhoods with the most affluent median neighborhood income. [26]

### Study Variables

The OCR provided information on age category (divided into 60 years and younger, 61–70, 71–80, and 80 years or older), sex, birth location (divided into people born in Canada and people born outside of Canada), cause of death, date of death, diagnosis date, postal code, and rural residence (rurality, classified by whether individuals were living in communities with less than 10,000 inhabitants [27]). Potentially curative treatment was considered as liver resection, liver transplantation, or radiofrequency ablation. Non-curative treatment was considered as chemotherapy or transarterial chemoembolization. Palliative treatment was considered as supportive treatment. Screening was considered as any ultrasound procedure one year prior to HCC diagnosis; patients were considered as being screened if they had more than one ultrasound within the last year of diagnosis; on the other hand, patients were not considered as being screened if they had only one ultrasound in the three months prior to diagnosis or if they were receiving HCC treatment or care prior to the ultrasound. Codes used to identify treatment and screening procedures can be found in Table S1.

Comorbidity score was calculated using the methods described by Charlson *et al*. [28] and Deyo *et al*., [29] applying an ICD-9 coding algorithm to the diagnostic field codes in our hospitalization data (excluding diagnoses for liver disease and metastatic cancer). Baseline comorbidity was determined using the hospitalization record at diagnosis date. Comorbidity involves assigning a severity value if any of a set of predetermined conditions appeared in any diagnosis field for a hospital episode. Based on the sum of these values for a given episode, it is then categorized into one of five groups (score 0, 1, 2, 3 or more, or no hospitalization record) representing different degrees of comorbidity. Thus, the score reflects both the number of comorbid conditions as well as their severity. If cases did not have a hospitalization record at diagnosis date, we determined baseline comorbidity by looking back two years into the hospitalization data to find the most recent hospitalization record and applying the comorbidity score from that hospitalization; 2,993 cases (55%) had a baseline hospitalization. Of the 2,488 patients (45%) who did not have a hospitalization at baseline, 1,405 (56%) had a hospitalization in the previous two years**.** Overall, 4,398 patients (80%) had a hospitalization date on diagnosis date or within the previous two years. Patients were assigned as having a missing comorbidity score at baseline if they had no hospitalization records at diagnosis or two years prior to diagnosis. Comorbidity was adjusted for each hospitalization after baseline.

### Statistical Analysis

Associations between SES (income quintile) and covariates were determined using chi-squared tests. We used logistic regression to measure the association between SES and comorbidity (Charlson Comorbidity score ≥1), receipt of screening, and receipt of curative treatment, after adjustment for age and sex. Median survival (months) with 95% confidence intervals (CI), 1-year survival, 2-year survival, and 5-year survival were analyzed by SES and other covariates using the Kaplan-Meier method. Differences in the survival between income quintile groups were assessed using the log-rank test. Cox proportional-hazards regression models were used to assess the association between income quintile and HCC survival. Our first set of Cox proportional hazard tests measured unadjusted hazard ratios for the explanatory variables. Our second set of Cox-proportional hazard tests measured the unadjusted and adjusted hazard ratios for the income quintiles, sequentially adjusting for age and sex, comorbidity, screening, and curative treatment. Age and sex were evaluated as confounders of SES, which was then followed by adding comorbidity, screening, and treatment to assess whether they were mediating variables of survival; finally, we adjusted for all covariates. Curative HCC treatment and comorbidity were modeled as time-dependent variables within the proportional-hazards regression models; treatment status changed from 0 to 1 based on the treatment date (if cases received curative treatment during illness); and Charlson comorbidity status of cases varied throughout follow-up based on the score from the previous hospitalization. In a separate analysis, we tested for potential effect modification between income quintile and receipt of ultrasound screening or curative treatment by including an interaction term in the fully adjusted model.

## Results

Among the diagnosed cancer cases in Ontario during the period 1990–2009, 5,481 cases were diagnosed principally as HCC. Table 1 shows the descriptive statistics of our cohort stratified by income quintiles. The majority of patients were males, with a male to female ratio of about 3∶1. The number of diagnosed HCC cases increased over the observation period, however, did not differ significantly between the income quintiles. Curative treatment for HCC was the only factor that was significantly different when stratified by income quintiles (p = 0.0003), with people from the lowest income quintile being less likely to receive curative treatment (25.3% for income quintile 1 vs. 30.5% to 32.6% for income quintiles 2–5). Non-curative treatment, palliative treatment, comorbidity, and ultrasound screening did not differ significantly between the income quintiles. After adjustment for age and sex (Table 2), comorbidity and screening were not significantly associated with income quintile; however, there was a strong association between income quintile and receipt of curative treatment (odds ratio [95%CI]: 1.53 [1.26–1.85] for income quintile 5 vs. quintile 1). Cases with missing income quintile information had statistically different characteristics when compared to the rest of the cohort on: rurality (p<0.0001), sex (p = 0.0049), country of birth (p<0.0001), curative treatment (p = 0.0003), non-curative treatment (p = 0.0245), palliative treatment (p<0.0001), HCC screening (p<0.0001), and index year diagnosed (p<0.0001). Descriptive statistics of the cohort by time period can be found in Table S2.

**Table 1:** Association of socioeconomic status with other potential prognostic variables among persons diagnosed with hepatocellular carcinoma, 1990–2009.

Variable	Income Quintile 1N (%)	Income Quintile 2N (%)	Income Quintile 3N (%)	Income Quintile 4N (%)	Income Quintile 5N (%)	MissingN (%)	p-value*
	N = 1323	N = 1196	N = 1030	N = 915	N = 893	N = 124	
Age group							
* 60 or below*	526 (39.7)	424 (35.4)	378 (36.7)	320 (35.0)	295 (33.0)	40 (32.3)	
* 61–70*	369 (27.9)	387 (32.4)	285 (27.7)	274 (30.0)	270 (30.2)	48 (38.7)	
* 71–80*	333 (25.2)	312 (26.1)	287 (27.9)	253 (27.6)	265 (29.7)	26 (21.0)	
* 81 or above*	95 (7.2)	73 (6.1)	80 (7.7)	68 (7.4)	63 (7.1)	10 (8.0)	0.0532
Sex (male)	1020 (77.1)	931 (77.8)	809 (78.5)	722 (78.9)	713 (79.8)	84 (67.7)	0.5988
Rurality							
* Urban residence*	1215 (91.8)	1110 (92.8)	937 (91.0)	831 (90.8)	821 (91.9)	16 (12.9)	
* Rural residence*	108 (8.2)	86 (7.2)	93 (9.0)	84 (9.2)	72 (8.1)	6 (4.8)	
* Missing*	0 (0.0)	0 (0.0)	0 (0.0)	0 (0.0)	0 (0.0)	102 (82.3)	0.4497
Country of birth							
* Canada*	423 (32.0)	388 (32.4)	343 (33.3)	306 (33.4)	325 (36.4)	60 (48.4)	
* Outside Canada*	582 (44.0)	500 (41.8)	421 (40.9)	374 (40.9)	331 (37.1)	51 (41.1)	
* Missing*	318 (24.0)	308 (25.8)	266 (25.8)	235 (25.7)	237 (26.5)	13 (10.5)	0.1766
Maximum Charlson Comorbidity score							
* 0*	499 (37.7)	444 (37.1)	383 (37.2)	350 (38.2)	334 (37.4)	54 (43.5)	
* 1*	290 (21.9)	264 (22.1)	202 (19.6)	182 (19.9)	182 (20.4)	29 (23.4)	
* 2*	165 (12.5)	128 (10.7)	130 (12.6)	123 (13.4)	114 (12.8)	15 (12.1)	
* 3 or more*	141 (10.7)	110 (9.2)	98 (9.5)	72 (7.9)	80 (9.0)	9 (7.3)	
* No hospitalization record*	228 (17.2)	250 (20.9)	217 (21.1)	188 (20.6)	183 (20.5)	17 (13.7)	0.3279
Screening with ultrasound 1 year prior to HCC diagnosis^†^	388 (29.3)	355 (29.7)	319 (31.0)	291 (31.8)	283 (31.7)	6 (4.8)	0.6148
HCC treatment^‡^							
* Curative*	335 (25.3)	365 (30.5)	336 (32.6)	294 (32.1)	288 (32.3)	19 (15.3)	0.0003
* Non-curative*	184 (13.9)	203 (17.0)	185 (18.0)	160 (17.5)	147 (16.5)	11 (8.9)	0.0637
* Palliative*	485 (36.7)	410 (34.3)	360 (35.0)	333 (36.4)	309 (34.6)	9 (7.3)	0.6798
* No treatment*	517 (39.1)	438 (36.6)	347 (33.8)	324 (35.4)	319 (35.7)	89 (71.8)	0.0936
Year of HCC diagnosis							
* 1990–1994*	156 (11.8)	146 (12.2)	111 (10.8)	94 (10.3)	104 (11.6)	105 (84.7)	
* 1995–1999*	252 (19.0)	239 (20.0)	196 (19.0)	180 (19.7)	172 (19.3)	- (3.2)	
* 2000–2004*	395 (29.9)	329 (27.5)	310 (30.1)	273 (29.8)	259 (29.0)	- (3.2)	
* 2005–2009*	520 (39.3)	482 (40.3)	413 (40.1)	368 (40.2)	358 (40.1)	11 (8.9)	0.9679

Income quintile 1, lowest socioeconomic status; Income quintile 5, highest socioeconomic status.

“-“, counts less than five have been suppressed. *p-values were calculated using chi-squared tests, testing for homogeneity across all income groups, excluding the missing groups. ^†^Patients were not considered as being screened if they had only one ultrasound in the three months prior to diagnosis or if they were receiving HCC care prior to the ultrasound. ^‡^Included multiple treatments for some people. HCC, hepatocellular carcinoma.

**Table 2:** Odds† of having a Charlson Comorbidity score greater than or equal to 1, receiving ultrasound screening, and receiving curative treatment, by income quintile.

Income quintile	Charlson Comorbidity score ≥1		Ultrasound screening^‡^		Potentially curative treatment	
	OR (95% CI)	p-value	OR (95% CI)	p-value	OR (95% CI)	p-value
1 (lowest)	1.00		1.00		1.00	
2	0.92 (0.78, 1.10)	0.381	1.02 (0.86, 1.21)	0.808	1.34 (1.12, 1.61)	0.001
3	0.91 (0.76, 1.10)	0.334	1.10 (0.92, 1.31)	0.315	1.51 (1.26, 1.82)	<0.001
4	0.88 (0.73, 1.06)	0.181	1.14 (0.95, 1.37)	0.159	1.49 (1.23, 1.80)	<0.001
5 (highest)	0.91 (0.75, 1.10)	0.305	1.14 (0.95, 1.37)	0.165	1.53 (1.26, 1.85)	<0.001

† Using logistic regression models adjusted for age and sex. ^‡^Patients were not considered as being screened if they had only one ultrasound in the three months prior to diagnosis or if they were receiving HCC care prior to the ultrasound.

Income quintile 1, lowest socioeconomic status; Income quintile 5, highest socioeconomic status.

OR, odds ratio; CI, confidence intervals.

**Figure 1:**
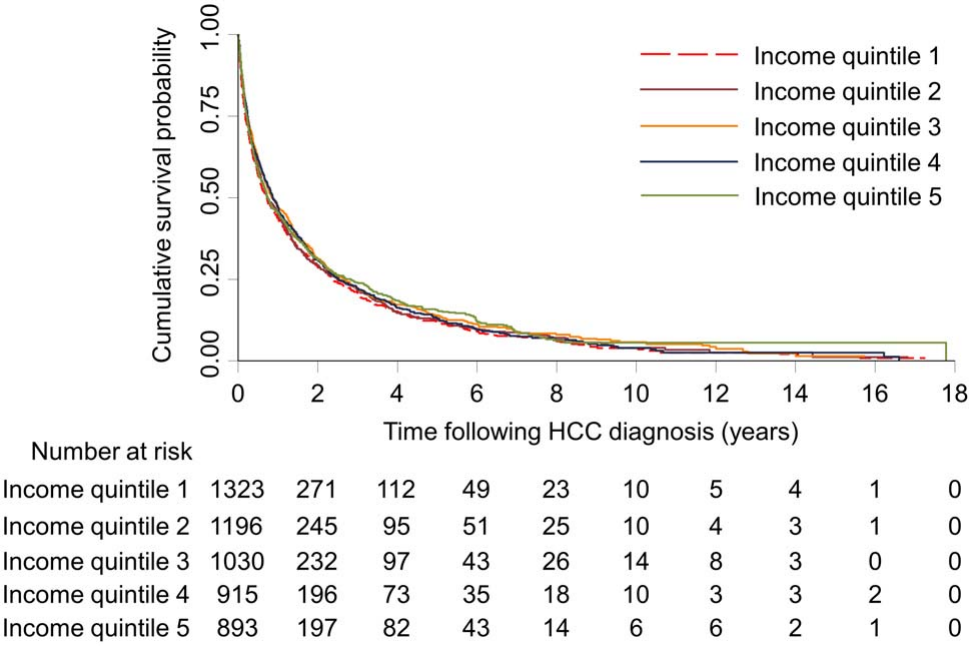
Kaplan-Meier survival estimates of people diagnosed with hepatocellular carcinoma by socio-economic status, 1990–2009. Log-rank test: p = 0.172. Income quintile 1, lowest socioeconomic status; Income quintile 5, highest socioeconomic status.

**Figure 2:**
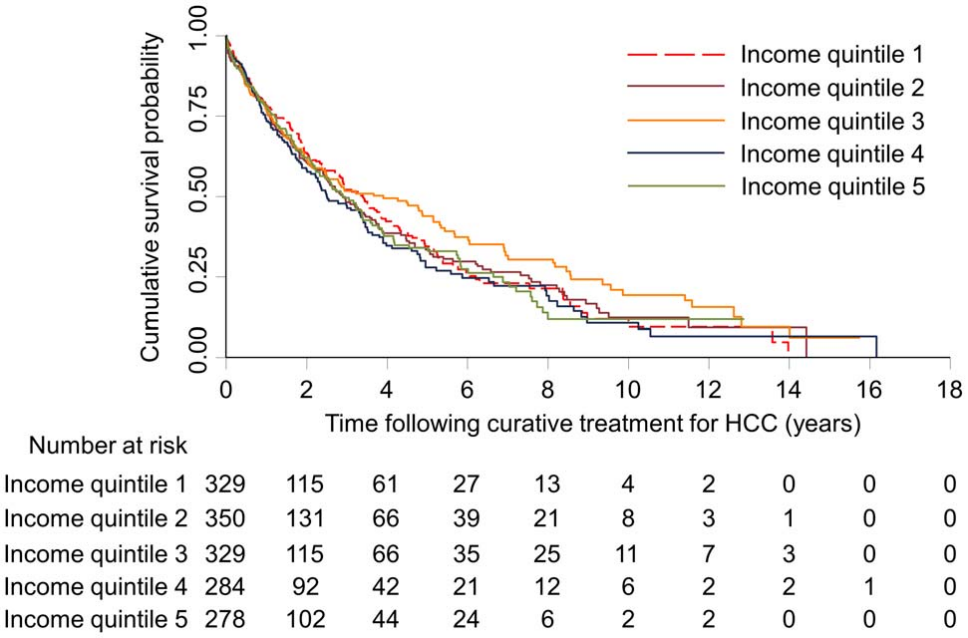
Kaplan-Meier survival estimates of people who received curative treatment for with hepatocellular carcinoma by socio-economic status, 1990–2009. Log-rank test: p = 0.376. Income quintile 1, lowest socioeconomic status; Income quintile 5, highest socioeconomic status.

Table 3 shows the median, 1-year, 2-year, and 5-year survival estimates. The overall median survival of the population was 9.2 months. The median survival estimates for income quintiles 1–5 were 8.5, 8.9, 10.5, 10.4, and 8.8 months, respectively. There was no significant difference in the overall survival between the income quintiles (log-rank test p = 0.172) (Fig. 1) or between the income quintiles of people who received curative treatment (log-rank test p = 0.376) (Fig. 2). Relative increases in median survival were found for patients who were younger (12.1 months for age 60 years or below vs. 5.5 months for age 81 years or above), had been screened using ultrasound anytime within one year prior to HCC diagnosis (19.0 months) vs. no screening (6.9 months), had received curative (44.4 months) or non-curative treatment (21.4 months) vs. no treatment (4.2 months), and had been diagnosed in more recent years (12.6 months for those diagnosed between 2005–2009 vs. 4.9 months for those diagnosed between 1990–1994). The observed increase in survival for people with higher comorbidity is likely a result of the fact that people who lived longer accumulated a greater maximal comorbidity score.

**Table 3:** Unadjusted survival of people diagnosed with hepatocellular carcinoma.

Characteristics	Cases	Events	Survival (Months)	1-Year Survival	2-Year Survival	5-Year Survival
	N (%)	N	Median (95% CI)	(%) (95% CI)	(%) (95% CI)	(%) (95% CI)
Overall	5481 (100)	4181	9.2 (8.7, 10.0)	45.2 (43.9, 46.6)	29.8 (28.5, 31.2)	13.2 (12.0, 14.3)
Income quintile						
* 1 (lowest)*	1323 (24.1)	1024	8.5 (7.3, 9.9)	43.5 (40.7, 46.3)	29.0 (26.3, 31.7)	11.9 (9.7, 14.1)
* 2*	1196 (21.8)	905	8.9 (7.9, 11.1)	44.9 (42.0, 47.9)	29.4 (26.5, 32.2)	12.6 (10.2, 14.9)
* 3*	1030 (18.8)	776	10.5 (8.8, 12.2)	47.2 (44.0, 50.4)	31.1 (28.0, 34.2)	13.8 (11.2, 16.5)
* 4*	915 (16.7)	692	10.4 (9.1, 12.2)	46.7 (43.3, 50.1)	30.6 (27.3, 33.9)	12.9 (10.1, 15.7)
* 5 (highest)*	893 (16.3)	667	8.8 (7.8, 10.6)	45.4 (42.0, 48.8)	30.9 (27.6, 34.2)	15.3 (12.3, 18.2)
Age group (years)						
* 60 or below*	1983 (36.2)	1325	12.1 (10.4, 13.9)	50.1 (47.7, 52.4)	35.6 (33.2, 38.0)	19.3 (17.0, 21.5)
* 61–70*	1633 (29.8)	1315	8.8 (7.9, 10.3)	43.9 (41.4, 46.4)	30.0 (27.6, 32.3)	13.3 (11.3, 15.3)
* 71–80*	1476 (26.9)	1208	8.6 (7.5, 9.9)	43.9 (41.2, 46.5)	26.2 (23.7, 28.6)	8.1 (6.3, 9.8)
* 81 or above*	389 (7.1)	333	5.5 (4.4, 7.0)	30.8 (26.0, 35.6)	15.4 (11.5, 19.3)	3.0 (0.7, 5.4)
Sex						
* Male*	4279 (78.1)	3247	9.1 (8.6, 10.0)	45.1 (43.5, 46.7)	30.0 (28.5, 31.5)	13.5 (12.2, 14.8)
* Female*	1202 (21.9)	934	9.4 (8.4, 11.3)	45.5 (42.5, 48.5)	29.1 (26.3, 31.9)	11.9 (9.6, 14.2)
Rurality						
* Urban residence*	4930 (89.9)	3714	9.6 (8.8, 10.4)	45.9 (44.4, 47.3)	30.5 (29.1, 31.9)	13.9 (12.7, 15.1)
* Rural residence*	449 (8.2)	369	8.3 (7.1, 10.0)	41.2 (37.4, 46.9)	25.8 (21.4, 30.2)	6.1 (3.3, 8.9)
Country of birth						
* Canada*	1845 (33.7)	1835	5.3 (4.8, 5.8)	30.5 (28.4, 32.6)	16.0 (14.3, 17.6)	3.8 (2.9, 4.7)
* Outside Canada*	2259 (41.2)	2247	6.5 (5.9, 7.0)	36.1 (34.1, 38.1)	19.9 (18.3, 22.6)	5.6 (4.6, 6.5)
Maximum Charlson Comorbidity score						
* 0*	2064 (37.7)	1638	5.4 (4.8, 6.0)	33.2 (30.9, 35.3)	20.1 (18.2, 22.0)	7.9 (6.4, 9.4)
* 1*	1149 (21.0)	847	8.9 (7.8, 10.8)	44.8 (41.8, 47.8)	29.7 (26.8, 32.6)	12.2 (9.7, 14.7)
* 2*	675 (12.3)	529	12.8 (10.8, 14.8)	52.0 (48.1, 55.9)	35.2 (31.4, 39.0)	18.1 (14.7, 21.5)
* 3 or more*	510 (9.3)	420	15.1 (12.3, 17.9)	55.3 (50.9, 59.7)	38.6 (34.1, 43.1)	15.5 (11.9, 19.1)
* No hospitalization record*	1083 (19.8)	747	16.3 (14.4, 18.4)	58.2 (55.1, 61.2)	39.4 (36.2, 42.6)	18.4 (15.4, 21.4)
Screening with ultrasound 1 year prior to HCC diagnosis†						
* No*	3839 (70.0)	3117	6.9 (6.4, 7.3)	38.2 (36.6, 39.8)	24.3 (22.8, 25.8)	10.1 (8.9, 11.3)
* Yes*	1642 (30.0)	1064	19.0 (17.2, 20.8)	62.0 (59.5, 64.5)	43.5 (40.8, 46.2)	20.8 (18.2, 23.4)
HCC treatment^‡^						
* Curative*	1637 (29.9)	766	44.4 (40.4, 46.9)	82.1 (80.1, 84.1)	68.1 (65.5, 70.7)	40.4 (37.2, 43.6)
* Non-curative*	890 (16.2)	627	21.4 (19.1, 23.0)	65.6 (62.4, 68.9)	45.1 (41.5, 48.6)	19.9 (16.8, 23.2)
* Palliative*	1906 (34.8)	1755	8.8 (8.1, 9.7)	42.7 (40.4, 45.0)	24.3 (22.3, 26.3)	7.8 (6.5, 9.1)
* No treatment*	2034 (37.1)	1765	4.2 (3.7, 4.6)	27.6 (25.6, 29.6)	15.4 (13.7, 17.1)	3.7 (2.7, 4.7)
Year of HCC diagnosis						
* 1990–1994*	716 (13.1)	672	4.9 (4.3, 5.8)	30.2 (26.8, 33.6)	17.6 (14.7, 20.5)	7.7 (5.7, 9.6)
* 1995–1999*	1043 (19.0)	953	6.2 (5.6, 7.6)	39.1 (36.1, 42.1)	26.9 (24.1, 29.6)	12.4 (10.3, 14.4)
* 2000–2004*	1570 (28.6)	1301	10.6 (9.3, 12.3)	48.1 (45.5, 50.6)	33.6 (31.5, 35.6)	15.0 (13.1, 16.9)
* 2005–2009*	2152 (39.3)	1255	12.6 (11.7, 13.8)	51.4 (49.1, 53.7)	32.1 (29.7, 34.6)	9.1 (3.4, 14.8)

† Patients were not considered as being screened if they had only one ultrasound in the three months prior to diagnosis or if they were receiving HCC care prior to the ultrasound. ^‡^Included multiple treatments for some people. CI, confidence intervals; HCC, hepatocellular carcinoma.

Table 4 shows the unadjusted Cox proportional-hazards model. The unadjusted proportional-hazards model for income quintiles showed that only income quintile 3 had a significantly different survival hazard ratio when compared to the lowest income group (income quintile 3 vs. quintile 1: unadjusted hazard ratio 0.899 [95% CI 0.819, 0.987]). In the adjusted model (Table 5), when income quintile was adjusted for age and sex, the higher income quintiles 3, 4, and 5 had approximately a 10% greater likelihood of survival compared to the lowest income quintile (income quintile 3 vs. quintile 1: adjusted hazard ratio 0.889 [95% CI 0.809, 0.976; income quintile 4 vs. quintile 1: adjusted hazard ratio 0.907 [95% CI 0.824, 0.999]; income quintile 5 vs. quintile 1: adjusted hazard ratio 0.905 [95% CI 0.821, 0.998]). Further adjustment of screening to the age- and sex-adjusted model did not affect the hazard ratio. When comorbidity was added to the age- and sex-adjusted model, the hazard ratios were affected slightly, with no survival advantage found among the higher income groups (income quintiles 4 and 5). When curative treatment was added to the age- and sex-adjusted model, the survival advantage of the higher income quintiles 3, 4, and 5 was no longer significant suggesting that higher rates of curative intent treatment among the higher income individuals explained their survival advantage. Notably, there was no significant interaction between SES and receiving ultrasound screening (p = 0.38) or receiving curative treatment (p = 0.75). When age, sex, comorbidity, and treatment were adjusted or in the completely adjusted model, no survival advantage was found among the higher income groups compared to the lowest income group.

**Table 4:** Risk of mortality after the diagnosis of hepatocellular carcinoma: unadjusted Cox proportional-hazards regression models.

Characteristic	Unadjusted Analysis		
	Hazard Ratio	95% CI	p-value
Income quintile			
* 1 (lowest)*	1 (referent)		
* 2*	0.954	0.872, 1.043	0.298
* 3*	0.899	0.819, 0.987	0.026
* 4*	0.922	0.837, 1.016	0.100
* 5 (highest)*	0.914	0.829, 1.007	0.070
Age group			
* 60 years or below*	1 (referent)		
* 61–70 years*	1.208	1.119, 1.304	<0.0001
* 71–80 years*	1.360	1.257, 1.470	<0.0001
* 81 years or above*	1.831	1.623, 2.066	<0.0001
Sex (male vs. female)	0.993	0.923, 1.068	0.844
Rurality			
* Urban residence*	1 (referent)		
* Rural residence*	1.184	1.064, 1.318	0.002
Country of birth			
* Canada*	1 (referent)		
* Outside Canada*	0.880	0.827, 0.936	<0.0001
Charlson Comorbidity score†			
* 0*	1 (referent)		
* 1*	1.209	1.115, 1.310	<0.0001
* 2*	1.865	1.680, 2.070	<0.0001
* 3 or more*	2.087	1.842, 2.365	<0.0001
* No hospitalization record*	0.146	0.114, 0.188	<0.0001
Screening with ultrasound 1 year prior to HCC diagnosis^‡^ (yes vs. no)	0.602	0.562, 0.646	<0.0001
HCC curative treatment† (yes vs. no)	0.379	0.349, 0.412	<0.0001
Year of HCC diagnosis			
* 1990–1994*	1 (referent)		
* 1995–1999*	0.837	0.758, 0.924	0.0004
* 2000–2004*	0.688	0.626, 0.756	<0.0001
* 2005–2009*	0.652	0.593, 0.717	<0.0001

† Variable modeled as time-dependent covariate. ^‡^Patients were not considered as being screened if they had only one ultrasound in the three months prior to diagnosis or if they were receiving HCC care prior to the ultrasound. CI, confidence intervals; HCC, hepatocellular carcinoma.

**Table 5:** Risk of mortality after the diagnosis of hepatocellular carcinoma: sequential analysis of the Cox proportional-hazards regression models.

Variables	Income quintile	Hazard Ratio (95% CI)	p-value
Unadjusted	1 (lowest)	1.00 (referent)	
	2	0.954 (0.872, 1.043)	0.298
	3	0.899 (0.819, 0.987)	0.026
	4	0.922 (0.837, 1.016)	0.100
	5 (highest)	0.914 (0.829–1.007)	0.070
Age and sex	1 (lowest)	1.00 (referent)	
	2	0.947 (0.866, 1.036)	0.237
	3	0.889 (0.809, 0.976)	0.013
	4	0.907 (0.824, 0.999)	0.048
	5 (highest)	0.905 (0.821, 0.998)	0.045
Age and sex + comorbidity†	1 (lowest)	1.00 (referent)	
	2	0.973 (0.890, 1.064)	0.547
	3	0.908 (0.827, 0.997)	0.042
	4	0.928 (0.842, 1.022)	0.127
	5 (highest)	0.917 (0.832, 1.011)	0.082
Age and sex + ultrasound screening^‡^	1 (lowest)	1.00 (referent)	
	2	0.936 (0.855, 1.023)	0.145
	3	0.879 (0.801, 0.965)	0.007
	4	0.906 (0.823, 0.998)	0.046
	5 (highest)	0.905 (0.821, 0.998)	0.045
Age and sex + curative treatment†	1 (lowest)	1.00 (referent)	
	2	0.997 (0.912, 1.091)	0.950
	3	0.938 (0.855, 1.030)	0.183
	4	0.937 (0.851, 1.032)	0.187
	5 (highest)	0.938 (0.850, 1.034)	0.198
Age and sex + comorbidity† + ultrasound screening^‡^	1 (lowest)	1.00 (referent)	
	2	0.961 (0.878, 1.051)	0.380
	3	0.900 (0.820, 0.988)	0.027
	4	0.922 (0.837, 1.016)	0.101
	5 (highest)	0.919 (0.834, 1.014)	0.092
Age and sex + comorbidity† + curative treatment†	1 (lowest)	1.00 (referent)	
	2	1.031 (0.942, 1.127)	0.506
	3	0.967 (0.881, 1.062)	0.484
	4	0.963 (0.874, 1.061)	0.445
	5 (highest)	0.949 (0.860, 1.046)	0.290
Age and sex + comorbidity† + ultrasound screening^‡^ + curative treatment†	1 (lowest)	1.00 (referent)	
	2	1.018 (0.930, 1.113)	0.703
	3	0.963 (0.877, 1.057)	0.430
	4	0.962 (0.873, 1.060)	0.435
	5 (highest)	0.952 (0.863, 1.050)	0.323
All variables	1 (lowest)	1.00 (referent)	
	2	1.038 (0.949, 1.136)	0.412
	3	0.953 (0.868, 1.047)	0.315
	4	0.968 (0.878, 1.066)	0.506
	5 (highest)	1.017 (0.922, 1.122)	0.729

† Variable modeled as time-dependent covariate. ^‡^Patients were not considered as being screened if they had only one ultrasound in the three months prior to diagnosis or if they were receiving HCC care prior to the ultrasound. CI, confidence intervals; HCC, hepatocellular carcinoma.

## Discussion

We sought to examine the influence of SES on the survival of HCC in Ontario. Our results indicate that there was a 10% survival advantage among the higher income quintile groups compared to the lowest income quintile group, after adjustment for age and sex. This association was no longer significant after adjustment for potentially curative treatment. Our findings suggest that individuals with higher SES have a survival advantage because they are more likely to receive curative treatment than those from lower SES strata.

Previous research has shown a clear association between SES and cancer, using many different SES indicators and in different settings. [6], [30] No study has previously analyzed the association of SES with HCC survival in Canada, although studies have been conducted in different countries and shown an association between HCC survival and SES. [4], [5], [8], [31], [32] One study conducted in Korea found an increased mortality risk of 72% in low income groups when comparing the highest SES group to the lowest, [31] while a second study conducted in Korea found a 57% increase in mortality when comparing high and low income groups, [32] Two studies performed in the US have also found an association between SES and HCC survival, identifying a 5% to 24% increased risk of mortality for low socioeconomic groups when compared to high socioeconomic groups. [5], [8]

Research from Ontario, Canada has shown some conflicting results regarding the association between SES and cancer survival. Gorey and colleagues [15], [16], [17], [18] have consistently shown no cancer survival gradient among SES groups in Ontario, in contrast to the survival advantage found for higher SES groups in the US. [15], [16], [17], [18], [33] The authors attribute the lack of association between cancer survival and SES in Ontario to the universal access to healthcare services, regardless of income. Equitable access to healthcare appears to compensate for any cancer survival advantage that people in higher SES might have. The observed cancer survival among the higher socioeconomic groups in the US on the other hand has been attributed to lack of equitable health service access.

Other studies by a different group of researchers have indicated a survival advantage for certain cancers among higher SES groups in Ontario. [12], [19], [20], [34] Cancers which have slower progression to metastatic cancer, are associated with symptoms that antedate advanced stage disease, and have a good prognosis have been found to have an association between survival and SES, whereas no association has been found between SES and cancer survival for cancers that have poor prognosis and in which symptoms are not identified before advanced stage. The explanation put forward for the observed association between cancer survival and SES in Ontario has been that people from higher SES seek medical attention earlier than people from lower SES if mild symptoms are present before the cancer becomes severe or incurable, leading to better prognosis, treatment, and survival among higher SES groups.

Our results contrast with previous studies. Although HCC is an aggressive cancer with very poor outcome, we found that inequities in outcome exist across SES groups, which appear to be due to differential rates of receiving potentially curative therapy. It is not clear why patients from lower SES groups are less likely to receive curative intent treatment. Although comorbidity was not statistically significant, individuals in the lowest income quintile were approximately 10% more likely to have a Charlson Comorbidity score greater than 1. Thus, it is possible that individuals with lower SES are more likely to be diagnosed at a more advanced stage disease at which point curative therapy is no longer possible. Unfortunately our data do not allow for determination of stage at diagnosis to clarify this issue. Notably, screening, which is critical to identification of HCC at a curable stage, was not associated with income level, even after adjustment for age and sex. Determination of the reasons for lower rates of curative treatment among lower income individuals is an important issue. In theory, the Canadian healthcare system should provide equal access to curative therapy for all individuals. It will be important to clarify whether SES impacts access to treatment directly, which seems relatively unlikely, or whether lower income individuals are more likely to present with more advanced disease precluding curative therapy. The latter explanation would be more in keeping with data on other cancers for which effective treatment relies on early diagnosis. Hopefully further investigation of the causes underlying our findings will lead to strategies to address the differences in rates of therapy and ultimately in outcome of HCC across income strata.

We acknowledge there are limitations to our study and the results should be interpreted with caution. Cases with missing SES information were significantly different than from the rest of the cohort. We postulate that the reason for the difference observed between cases with missing SES information and the rest of the cohort is due to the fact that the majority of cases with missing SES information were from the 1990–1994 cohort period, which had significantly lower screening rates, treatment procedures, and different population demographics compared to other years. Median neighborhood household income, an economic surrogate marker of a patient’s financial status, was used as our main variable for SES, due to the lack of individual level SES data. Since an economic surrogate marker was used for SES, associations between SES and cancer survival may have been attenuated and less sensitive towards revealing the true relationship. [35], [36] Although our results are subject to misclassification bias, economic surrogate markers of SES have been validated as a proxy variable for individual level data, and found to be highly correlated with other socioeconomic indicators; [36], [37] therefore we believe our results reflect true associations. We were unable to account for some potential confounders such as the stage of HCC at diagnosis and clinical information due to limitations of data within the cancer registry. To compensate for lack of stage data, we included individual comorbidity data, providing some measure of the degree of illness within the SES groups.

In conclusion, our population-based study suggests that there is a relationship between SES and HCC survival in Ontario. This association is most likely explained due to difference in curative treatment rates among the income groups, where lower income groups are less likely to receive potentially curative treatment. Although there may be other contributors, our data suggest that access to curative therapy or presentation with later stage disease are the most likely causes of health disparities related to SES. Further research should focus on determining the barriers to access either to curative therapy or to early diagnosis of HCC in Canada.

## Supporting Information

Table S1 Treatment and screening procedures for people with hepatocellular carcinoma
(DOC)

Table S2 Descriptive statistics of the cohort by time period, 1990–2009
(DOC)
