# Supplementary material for: Influence of Socioeconomic Status on Survival of Hepatocellular Carcinoma in the Ontario Population; A Population-Based Study, 1990–2009
Source: PLoS One. 2012 Jul 13;7(7):e40917. doi: 10.1371/journal.pone.0040917 (PMC3396620; doi:10.1371/journal.pone.0040917)
Supplement: Table S1 — Treatment and screening procedures for people with hepatocellular carcinoma (DOC) [file pone.0040917.s001.doc]

**Supporting Information**

Table S1. Treatment and screening procedures for people with hepatocellular carcinoma

|  | CCP code | CCI code | OHIP code |
| --- | --- | --- | --- |
| **Treatment** |  |  |  |
| *Potentially curative therapy* |  |  |  |
| Local excision or destruction of lesion or tissue of liver | 62.1 |  |  |
| Partial hepatectomy | 62.12 |  |  |
| Other destruction of lesion of liver | 62.19 |  |  |
| Lobectomy of liver | 62.20 |  |  |
| Excision partial, liver using endoscopic (laparoscopic)approach |  | 1OA87DA |  |
| Excision partial, liver using open approach |  | 1OA87LA |  |
| Excision partial, liver using ultrasonic aspirator device (for dissection) and open approach |  | 1OA87LAAZ |  |
| Liver excision-complete left/right lobectomy |  |  | S267 |
| Liver excision-of lesion |  |  | S269 |
| Liver excision-hepatectomy left lateral segmental excision |  |  | S270 |
| Liver excision-extended right lobectomy |  |  | S271 |
| Liver excision-partial lobectomy |  |  | S275 |
| Total hepatectomy | 62.3 |  |  |
| Liver transplant | 62.4 |  |  |
| Auxiliary liver transplant | 62.41 |  |  |
| Other transplant of liver | 62.49 |  |  |
| Transplant, liver of a deceased donor full size liver |  | 1OA85LAXXK |  |
| Transplant, liver of a deceased donor multi organ liver with intestine/ pancreas/spleen/stomach (or any combination of) |  | 1OA85VCXXK |  |
| Transplant, liver of a living donor split liver |  | 1OA85WLXXJ |  |
| Transplant, liver of a deceased donor split liver (or reduced paediatric-size liver) |  | 1OA85WLXXK |  |
| Living donor orthotopic liver transplantation recipient |  |  | S266 |
| Liver excision-liver transplant-recipient |  |  | S294 |
| Digestive system-liver-repeat liver transplant |  |  | S295 |
| Destruction, liver endoscopic (laparoscopic) approach using radiofrequency |  | 1OA59DAAW |  |
| Destruction, liver percutaneous approach using radiofrequency |  | 1OA59HAAW |  |
| Destruction, liver open approach using radiofrequency |  | 1OA59LAAW |  |
| Radiofrequency ablation |  |  | J069 |
| *Non-curative therapy* |  |  |  |
| Percutaneous ablation |  |  |  |
| Destruction, liver endoscopic (abdominal) approach using cryoprobe |  | 1OA59DAAD |  |
| Destruction, liver endoscopic (abdominal) approach using laser |  | 1OA59DAAG |  |
| Destruction, liver endoscopic (abdominal) approach using device NEC |  | 1OA59DAGX |  |
| Destruction, liver endoscopic (abdominal) approach using chemical cautery agent (e.g. ethanol) |  | 1OA59DAX7 |  |
| Destruction, liver percutaneous approach using chemical cautery agent (e.g. ethanol) |  | 1OA59HAX7 |  |
| Destruction, liver open approach using cryoprobe |  | 1OA59LAAD |  |
| Destruction, liver open approach using laser |  | 1OA59LAAG |  |
| Destruction, liver open approach using device NEC |  | 1OA59LAGX |  |
| Destruction, liver open approach using chemical cautery agent (e.g. ethanol) |  | 1OA59LAX7 |  |
| Chemotherapy |  |  |  |
| Diagnostic and therapeutic injection(s)/infusion(s) test dose (bleomycin&l-asparatiginase once per patient per drug) |  |  | G075 |
| Diagnostic and therapeutic injection/infusion-intravenous chemotherapy- each additional injection to |  |  | G281 |
| Single agent intravenous chemotherapy i.e. doxorubicin, daunorubicin, epirubicin, mitoxintrone, cisplatin or bleomycin (greater than 10 units per metre square) |  |  | G339 |
| Taxol, rituximab, trastuzumab, bortezomib, docetaxel administration or multiple agent intravenous chemotherapy including at least one of either doxorubicin, daunorubicin, epirubicin, mitoxintrone, cisplatin or bleomycin (greater than 10 units per metre square) |  |  | G345 |
| Special single agent chemotherapy utilizing either high-dose methotrexate with folinic acid rescue - methotrexate given in a dose of greater than 1 g/m2, high dose cisplatin greater than 75 mg/m2 given concurrently with hydration and osmotic diuresis, high dose cystosine, arabinoside (greater than 2g/m2), or high dose cyclophosphamide (greater than 1g/m2) |  |  | G359 |
| Single injection (for agents other than doxorubicin, cisplatin,  bleomycin or high dose methotrexate) |  |  | G381 |
| Supervision of chemotherapy (marrow suppressant) for  malignant or autoimmune disease by telephone - monthly |  |  | G382 |
| Arteries-cannulation-chemotherapy-hepatic (TACE) |  |  | R776 |
| *Supportive/Palliative care* |  |  |  |
| General/Family Practice special palliative care consultation |  |  | A945 |
| Special palliative care consultation hospital in patient |  |  | C945 |
| Palliative care |  |  | C982 |
| Palliative care support individual care 1/2 hr. or major part |  |  | K023 |
| **Screening** |  |  |  |
| Diagnostic ultrasound-abdomen-abdominal scan-limited study |  |  | J128 |
| Diagnostic ultrasound-abdomen/retroperitoneal abdominal scan complete |  |  | J135 |
| Diagnostic ultrasound-abdomen & retroperitoneal.p2-abdominal scan-limited study |  |  | J428 |
| Diagnostic ultrasound- abdomen & retroperitoneal.p2-abdominal scan complete |  |  | J435 |

CCI, Canadian Classification of Health Interventions – is the new national standard for classifying health care procedures. CCI is the companion classification system to ICD-10-CA. CCI replaces the Canadian Classification of Diagnostic, Therapeutic and Surgical Procedures (CCP) and the intervention portion of ICD-9-CM in Canada. CCP was originally developed by Statistics Canada in 1978 to meet Canadian needs for a procedural classification to be used in conjunction with ICD-9.
