# Supplementary material for: Influence of Socioeconomic Status on Survival of Hepatocellular Carcinoma in the Ontario Population; A Population-Based Study, 1990–2009
Source: PLoS One. 2012 Jul 13;7(7):e40917. doi: 10.1371/journal.pone.0040917 (PMC3396620; doi:10.1371/journal.pone.0040917)
Supplement: Table S2 — Descriptive statistics of the cohort by time period, 1990–2009 (DOC) [file pone.0040917.s002.doc]

**Supporting Information**

Table S2. Descriptive statistics of the cohort by time period, 1990-2009

| Time period | Variable | Income Quintile 1 | Income Quintile 2 | Income Quintile 3 | Income Quintile 4 | Income Quintile 5 | Missing |
| --- | --- | --- | --- | --- | --- | --- | --- |
| 1990-1994 |  | N = 156 | N = 146 | N = 111 | N = 94 | N = 104 | N=105 |
|  | Age group |  |  |  |  |  |  |
|  | *60 or below* | 61 (39.1) | 53 (36.3) | 43 (38.7) | 26 (27.7) | 34 (32.7) | 35 (33.3) |
|  | *61-70* | 53 (34.0) | 65 (44.2) | 38 (34.2) | 40 (42.6) | 32 (30.8) | 41 (39.1) |
|  | *71-80* | 35 (22.4) | 24 (16.4) | 20 (18.0) | 23 (24.5) | 32 (30.8) | 21 (20.0) |
|  | *81 or above* | 7 (4.5) | - (2.7) | 10 (9.0) | 5 (5.3) | 6 (5.8) | 8 (7.6) |
|  | Sex (male) | 118 (75.6) | 113 (77.4) | 85 (76.8) | 69 (73.4) | 82 (78.9) | 72 (68.6) |
|  | Rurality |  |  |  |  |  |  |
|  | *Urban residence* | 148 (94.9) | 136 (93.2) | 98 (88.3) | 79 (84.0) | 95 (91.4) | 7 (6.7) |
|  | *Rural residence* | 8 (5.1) | 10 (6.9) | 13 (11.7) | 15 (16.0) | 9 (8.6) | - (1.9) |
|  | *Missing* | 0 (0.0) | 0 (0.0) | 0 (0.0) | 0 (0.0) | 0 (0.0) | 96 (91.4) |
|  | Country of birth |  |  |  |  |  |  |
|  | *Canada* | 48 (30.7) | 51 (34.9) | 40 (36.6) | 34 (36.2) | 41 (39.4) | 51 (48.6) |
|  | *Outside Canada* | 97 (62.2) | 81 (55.5) | 58 (52.3) | 56 (59.6) | 58 (55.8) | 50 (47.6) |
|  | *Missing* | 11 (7.1) | 14 (9.6) | 13 (11.7) | - (4.3) | 5 (4.8) | - (3.8) |
|  | Maximum Charlson Comorbidity score |  |  |  |  |  |  |
|  | *0* | 76 (48.7) | 70 (48.0) | 49 (44.1) | 46 (48.9) | 48 (46.2) | 49 (46.7) |
|  | *1* | 33 (21.2) | 33 (22.6) | 29 (26.1) | 19 (20.2) | 25 (24.0) | 23 (21.9) |
|  | *2* | 21 (13.5) | 13 (8.9) | 15 (13.5) | 13 (13.8) | 18 (17.3) | 12 (11.4) |
|  | *3 or more* | 10 (6.4) | 8 (5.5) | 6 (5.4) | - (2.1) | - (1.9) | 6 (5.7) |
|  | *No hospitalization record* | 16 (10.3) | 22 (15.1) | 12 (10.8) | 14 (14.9) | 11 (10.6) | 15 (14.3) |
|  |  |  |  |  |  |  |  |
|  | Screening with ultrasound 1 year prior to HCC diagnosis^†^ | 16 (10.3) | 24 (16.4) | 13 (11.7) | 11 (11.7) | 21 (20.2) | - (2.9) |
|  | HCC treatment^‡^ |  |  |  |  |  |  |
|  | *Curative* | 29 (18.6) | 27 (18.5) | 31 (27.9) | 19 (20.2) | 19 (18.3) | 18 (17.1) |
|  | *Non-curative* | 29 (18.6) | 22 (15.1) | 21 (18.9) | 12 (12.8) | 17 (16.4) | 7 (6.7) |
|  | *Palliative* | 27 (17.3) | 19 (13.0) | 14 (12.6) | 12 (12.8) | 13 (12.5) | - (2.9) |
|  | *No treatment* | 91 (58.3) | 83 (56.9) | 60 (54.1) | 59 (62.8) | 65 (62.5) | 79 (75.2) |

Income quintile 1, lowest socioeconomic status; Income quintile 5, highest socioeconomic status.

“-“, counts less than five have been suppressed. ^†^Patients were not considered as being screened if they had only one ultrasound in the three months prior to diagnosis or if they were receiving HCC care prior to the ultrasound. ^‡^Included multiple treatments for some people. HCC, hepatocellular carcinoma.

Table S2. Descriptive statistics of the cohort by time period, 1990-2009 (continued)

| Time period | Variable | Income Quintile 1 | Income Quintile 2 | Income Quintile 3 | Income Quintile 4 | Income Quintile 5 | Missing |
| --- | --- | --- | --- | --- | --- | --- | --- |
| 1995-1999 |  | N = 252 | N = 239 | N = 196 | N = 180 | N = 172 | - |
|  | Age group |  |  |  |  |  |  |
|  | *60 or below* | 88 (34.9) | 74 (31.0) | 66 (33.7) | 64 (35.6) | 43 (25.0) | - (25.0) |
|  | *61-70* | 79 (31.4) | 90 (37.7) | 68 (34.7) | 56 (31.1) | 72 (41.9) | - (25.0) |
|  | *71-80* | 76 (30.2) | 62 (25.9) | 51 (26.0) | 52 (28.9) | 44 (25.6) | - (50.0) |
|  | *81 or above* | 9 (3.6) | 13 (5.4) | 11 (5.6) | 8 (4.4) | 13 (7.6) | 0 (0.0) |
|  | Sex (male) | 194 (77.0) | 182 (76.2) | 158 (80.6) | 144 (80.0) | 136 (79.1) | - (75.0) |
|  | Rurality |  |  |  |  |  |  |
|  | *Urban residence* | 227 (90.1) | 218 (91.2) | 181 (92.4) | 160 (88.9) | 165 (95.9) | 0 (0.0) |
|  | *Rural residence* | 25 (9.9) | 21 (8.8) | 15 (7.7) | 20 (11.1) | 7 (4.1) | - (25.0) |
|  | *Missing* | 0 (0.0) | 0 (0.0) | 0 (0.0) | 0 (0.0) | 0 (0.0) | - (75.0) |
|  | Country of birth |  |  |  |  |  |  |
|  | *Canada* | 87 (34.5) | 83 (34.7) | 85 (43.4) | 80 (44.4) | 67 (39.0) | - (50.0) |
|  | *Outside Canada* | 136 (54.0) | 137 (57.3) | 93 (47.5) | 92 (51.1) | 84 (48.8) | 0 (0.0) |
|  | *Missing* | 29 (11.5) | 19 (8.0) | 18 (9.2) | 8 (4.4) | 21 (12.2) | - (50.0) |
|  | Maximum Charlson Comorbidity score |  |  |  |  |  |  |
|  | *0* | 110 (43.7) | 97 (40.6) | 79 (40.3) | 68 (37.8) | 76 (44.2) | 0 (0.0) |
|  | *1* | 52 (20.6) | 56 (23.4) | 36 (18.4) | 40 (22.2) | 34 (19.8) | - (25.0) |
|  | *2* | 28 (11.1) | 24 (10.0) | 30 (15.3) | 29 (16.1) | 17 (9.9) | - (25.0) |
|  | *3 or more* | 22 (8.7) | 19 (8.0) | 23 (11.7) | 15 (8.3) | 15 (8.7) | - (25.0) |
|  | *No hospitalization record* | 40 (15.9) | 43 (18.0) | 28 (14.3) | 28 (15.6) | 30 (17.4) | - (25.0) |
|  | Screening with ultrasound 1 year prior to HCC diagnosis^†^ | 61 (24.2) | 71 (29.7) | 43 (21.9) | 48 (26.7) | 47 (27.3) | 0 (0.0) |
|  | HCC treatment^‡^ |  |  |  |  |  |  |
|  | *Curative* | 48 (19.1) | 57 (23.9) | 46 (23.5) | 44 (24.4) | 54 (31.4) | 0 (0.0) |
|  | *Non-curative* | 20 (7.9) | 30 (12.6) | 34 (17.4) | 26 (14.4) | 16 (9.3) | - (25.0) |
|  | *Palliative* | 78 (31.0) | 63 (26.4) | 58 (29.6) | 66 (36.7) | 43 (25.00) | 0 (0.0) |
|  | *No treatment* | 136 (54.0) | 122 (51.1) | 90 (45.9) | 81 (45.0) | 84 (48.8) | - (75.0) |

Table S2. Descriptive statistics of the cohort by time period, 1990-2009 (continued)

| Time period | Variable | Income Quintile 1 | Income Quintile 2 | Income Quintile 3 | Income Quintile 4 | Income Quintile 5 | Missing |
| --- | --- | --- | --- | --- | --- | --- | --- |
| 2000-2004 |  | N = 395 | N = 329 | N = 310 | N = 273 | N = 259 | - |
|  | Age group |  |  |  |  |  |  |
|  | *60 or below* | 156 (39.5) | 105 (31.9) | 124 (40.0) | 91 (33.3) | 102 (39.4) | - (50.0) |
|  | *61-70* | 124 (31.4) | 107 (32.5) | 75 (24.2) | 86 (31.5) | 62 (23.9) | - (50.0) |
|  | *71-80* | 90 (22.8) | 96 (29.2) | 93 (30.0) | 80 (29.3) | 80 (30.9) | 0 (0.0) |
|  | *81 or above* | 25 (6.3) | 21 (6.4) | 18 (5.8) | 16 (5.9) | 15 (5.8) | 0 (0.0) |
|  | Sex (male) | 304 (77.0) | 246 (74.8) | 242 (78.1) | 218 (79.9) | 208 (80.3) | - (75.0) |
|  | Rurality |  |  |  |  |  |  |
|  | *Urban residence* | 363 (91.9) | 302 (91.8) | 289 (93.2) | 252 (92.3) | 240 (92.7) | - (50.0) |
|  | *Rural residence* | 32 (8.1) | 27 (8.2) | 21 (6.8) | 21 (7.7) | 19 (7.3) | - (25.0) |
|  | *Missing* | 0 (0.0) | 0 (0.0) | 0 (0.0) | 0 (0.0) | 0 (0.0) | -(25.0) |
|  | Country of birth |  |  |  |  |  |  |
|  | *Canada* | 133 (33.7) | 123 (37.4) | 103 (33.3) | 97 (35.5) | 104 (40.2) | - (50.0) |
|  | *Outside Canada* | 203 (51.4) | 148 (45.0) | 147 (47.4) | 130 (47.6) | 106 (40.9) | 0 (0.0) |
|  | *Missing* | 59 (14.9) | 58 (17.6) | 60 (19.4) | 46 (16.9) | 49 (18.9) | - (50.0) |
|  | Maximum Charlson Comorbidity score |  |  |  |  |  |  |
|  | *0* | 150 (38.0) | 109 (33.1) | 115 (37.1) | 101 (37.0) | 93 (35.9) | - (50.0) |
|  | *1* | 79 (20.0) | 73 (22.2) | 59 (19.0) | 53 (19.4) | 48 (18.5) | - (25.0) |
|  | *2* | 50 (12.7) | 40 (12.1) | 32 (10.3) | 39 (14.3) | 32 (12.4) | - (25.0) |
|  | *3 or more* | 50 (12.7) | 38 (11.6) | 31 (10.0) | 22 (8.1) | 21 (8.1) | 0 (0.0) |
|  | *No hospitalization record* | 66 (16.7) | 69 (21.0) | 73 (23.6) | 58 (21.3) | 65 (25.1) | 0 (0.0) |
|  | Screening with ultrasound 1 year prior to HCC diagnosis^†^ | 129 (32.7) | 93 (28.3) | 101 (32.6) | 105 (38.5) | 81 (31.3) | 0 (0.0) |
|  | HCC treatment^‡^ |  |  |  |  |  |  |
|  | *Curative* | 106 (26.8) | 104 (31.6) | 110 (35.5) | 90 (33.0) | 89 (34.4) | 0 (0.0) |
|  | *Non-curative* | 62 (15.7) | 56 (17.0) | 50 (16.1) | 51 (18.7) | 46 (17.8) | - (25.0) |
|  | *Palliative* | 172 (43.5) | 140 (42.6) | 121 (39.0) | 124 (45.4) | 118 (45.6) | - (25.0) |
|  | *No treatment* | 133 (33.7) | 102 (31.0) | 96 (31.0) | 86 (31.5) | 74 (28.6) | - (50.0) |

Table S2. Descriptive statistics of the cohort by time period, 1990-2009 (continued)

| Time period | Variable | Income Quintile 1 | Income Quintile 2 | Income Quintile 3 | Income Quintile 4 | Income Quintile 5 | Missing |
| --- | --- | --- | --- | --- | --- | --- | --- |
| 2005-2009 |  | N = 520 | N = 482 | N =413 | N = 368 | N = 358 | N=11 |
|  | Age group |  |  |  |  |  |  |
|  | *60 or below* | 221 (42.5) | 192 (39.8) | 145 (35.1) | 139 (37.8) | 116 (32.4) | - (18.2) |
|  | *61-70* | 113 (21.7) | 125 (25.9) | 104 (25.2) | 92 (25.0) | 104 (29.1) | - (36.4) |
|  | *71-80* | 132 (25.4) | 130 (27.0) | 123 (29.8) | 98 (26.6) | 109 (30.5) | - (27.3) |
|  | *81 or above* | 54 (10.4) | 35 (7.3) | 41 (9.9) | 39 (10.6) | 29 (8.1) | - (18.2) |
|  | Sex (male) | 404 (77.7) | 390 (80.9) | 324 (78.5) | 291 (79.1) | 287 (80.2) | 6 (54.6) |
|  | Rurality |  |  |  |  |  |  |
|  | *Urban residence* | 477 (91.7) | 454 (94.2) | 369 (89.4) | 340 (92.4) | 321 (89.7) | 7 (63.6) |
|  | *Rural residence* | 43 (8.3) | 28 (5.8) | 44 (10.7) | 28 (7.6) | 37 (10.3) | - (18.2) |
|  | *Missing* | 0 (0.0) | 0 (0.0) | 0 (0.0) | 0 (0.0) | 0 (0.0) | - (18.2) |
|  | Country of birth |  |  |  |  |  |  |
|  | *Canada* | 155 (29.8) | 131 (27.2) | 115 (27.9) | 95 (25.8) | 113 (31.7) | 5 (45.5) |
|  | *Outside Canada* | 146 (28.1) | 134 (27.8) | 123 (29.8) | 96 (26.1) | 83 (23.2) | - (9.1) |
|  | *Missing* | 219 (42.1) | 217 (45.0) | 175 (42.4) | 177 (48.1) | 162 (42.3) | 5 (45.5) |
|  | Maximum Charlson Comorbidity score |  |  |  |  |  |  |
|  | *0* | 163 (31.4) | 168 (34.9) | 140 (33.9) | 135 (36.7) | 117 (32.9) | - (27.3) |
|  | *1* | 126 (24.2) | 102 (21.2) | 78 (18.9) | 70 (19.0) | 75 (21.0) | - (36.4) |
|  | *2* | 66 (12.7) | 51 (10.6) | 53 (12.8) | 42 (11.4) | 47 (13.1) | - (18.2) |
|  | *3 or more* | 59 (11.4) | 45 (9.3) | 38 (9.2) | 33 (9.0) | 42 (11.7) | - (9.1) |
|  | *No hospitalization record* | 106 (20.4) | 116 (24.1) | 104 (25.2) | 88 (23.9) | 77 (21.5) | - (9.1) |
|  | Screening with ultrasound 1 year prior to HCC diagnosis^†^ | 182 (35.0) | 167 (34.7) | 162 (39.2) | 127 (34.5) | 134 (37.4) | - (27.3) |
|  | HCC treatment^‡^ |  |  |  |  |  |  |
|  | *Curative* | 152 (29.2) | 177 (36.7) | 149 (36.1) | 141 (38.3) | 126 (35.2) | - (9.1) |
|  | *Non-curative* | 73 (14.0) | 95 (19.7) | 80 (19.4) | 71 (19.3) | 68 (19.0) | - (18.2) |
|  | *Palliative* | 208 (40.0) | 188 (39.0) | 167 (40.4) | 131 (35.6) | 135 (37.7) | 5 (45.5) |
|  | *No treatment* | 157 (30.2) | 131 (27.2) | 101 (24.5) | 98 (26.6) | 96 (26.8) | 5 (45.5) |
